# Characterization and Functional Analysis of a Type 2 Diacylglycerol Acyltransferase (*DGAT2*) Gene from Oil Palm (*Elaeis guineensis* Jacq.) Mesocarp in *Saccharomyces cerevisiae* and Transgenic *Arabidopsis thaliana*

**DOI:** 10.3389/fpls.2017.01791

**Published:** 2017-10-17

**Authors:** Yuanhang Jin, Yijun Yuan, Lingchao Gao, Ruhao Sun, Lizhi Chen, Dongdong Li, Yusheng Zheng

**Affiliations:** Key Laboratory of Tropical Biological Resources of Ministry of Education, College of Materials and Chemical Engineering, Hainan University, Haikou, China

**Keywords:** oil palm, oil synthesis, diacylglycerol acyltransferase, *Arabidopsis thaliana*, *Saccharomyces cerevisiae*

## Abstract

Oil palm (*Elaeis guineensis* Jacq.) is the highest oil-yielding plant in the world, storing 90 and 60% (dry weight) oil in its mesocarp and kernel, respectively. To gain insights into the oil accumulation mechanism, one of the key enzymes involved in triacylglycerol (TAG) biosynthesis, a Type 2 diacylglycerol acyltransferase (DGAT2) from oil palm, was characterized for its *in vivo* activity. *EgDGAT2* is highly expressed in mesocarp during the last two developmental stages while large amounts of oil are accumulated at the highest rate during ripening. Heterologous expression of *EgDGAT2* in mutant yeast H1246 restored TAG biosynthesis with substrate preference toward unsaturated fatty acids (FAs) (16:1 and 18:1). Furthermore, seed-specific overexpression of *EgDGAT2* in *Arabidopsis thaliana* enhanced the content of polyunsaturated FAs 18:2 and 18:3 (each by 6 mol%) in seed TAGs, when compared to that from wild-type Arabidopsis. In turn, the proportion of 18:0 and 20:0 FAs in seed TAGs from EgDGAT2 transgenic lines decreased accordingly. These results provide new insights into understanding the *in vivo* activity of EgDGAT2 from oil palm mesocarp, which will be of importance for metabolic enhancement of unsaturated FAs production.

## Introduction

Plant oils, mainly in the form of triacylglycerols (TAGs), are important renewable sources for food and feed and, increasingly, as feedstocks for biodiesel and industrial chemicals (Dyer et al., 2008; Xu and Shanklin, 2016). TAGs, as the major storage lipids, are the most abundant energy-dense storage compounds primarily accumulated in seeds and fruits. The value and applications of TAGs are largely determined by three esterified fatty acids (FAs) with chain lengths of C8–C24, which vary greatly among species (Dyer et al., 2008). The five most common FAs are saturated palmitic acid (16:0) and stearic acid (18:0), monounsaturated oleic acid (18:1), and polyunsaturated linoleic acid (18:2) and linolenic acid (18:3) (Bates and Browse, 2012).

In plants, oil biosynthesis consists of *de novo* FA synthesis and TAG assembly, occurring in the plastid and endoplasmic reticulum (ER), respectively. The nascent FAs are synthesized by FAsynthase (FAS), and then exported into the ER for TAG assembly. The simplest way to produce TAGs is via the Kennedy pathway, involving sequential acylation of the glycerol backbone with three *sn*-specific acyltransferases (Bates and Browse, 2012; Li-Beisson et al., 2013). First, two sequential acylations at the *sn*-1 and *sn*-2 positions of glycerol-3-phosphate with acyl-CoA lead to synthesis of lysophosphatidic acid and subsequently phosphatidic acid (PA) by glycerol-3-phosphate acyltransferase and lysophosphatidic acid acyltransferase, respectively (Bates and Browse, 2012). Then, PA is dephosphorylated to diacylglycerol (DAG) by PA phosphatase. Both PA and DAG are common precursors for phospholipids and TAGs (Chapman and Ohlrogge, 2012). Finally, TAGs are synthesized by the third acylation of acyl-CoAs at the *sn*-3 position of DAG molecules by diacylglycerol acyltransferase (DGAT). Therefore, DGAT catalyzes the only step exclusively committed to TAG synthesis and plays a key role in determining the carbon flux into TAGs (Liu et al., 2012).

Until now, four types of DGATs have been identified in higher plants based on structure and activity. Two main classes of DGAT, designated Type 1 DGAT (DGAT1) and Type DGAT (DGAT2), are ubiquitous in eukaryotes (Liu et al., 2012). They are two structurally distinct groups of membrane-bound acyltransferases and have no sequence homology. In addition, they differ in intracellular localization, substrate specificity, and expression pattern (Shockey et al., 2006; Liu et al., 2012; Li-Beisson et al., 2013). DGAT1s are predicted to contain between 8 and 10 transmembrane domains and belong to the membrane-bound *O*-acyltransferase family, whereas DGAT2 polypeptides have only two to three predicted transmembrane domains and belong to the monoacylglycerol acyltransferase family (Liu et al., 2012; Chen et al., 2016). A third soluble class of DGAT enzyme has been reported in peanut and Arabidopsis (Saha et al., 2006; Hernandez et al., 2012). A fourth type of DGAT enzymes are wax ester synthase (WS)/DGAT homologs to a bifunctional membrane-associated enzyme identified from *Acinetobacter calcoaceticus* ADP1 that possesses both WS and DGAT activities (Kalscheuer and Steinbüchel, 2003; Li et al., 2008). However, WS/DGAT homologs identified from Arabidopsis and petunia (*Petunia hybrida*) predominantly catalyzed the synthesis of wax esters (King et al., 2007; Li et al., 2008).

Type 1 diacylglycerol acyltransferase (DGAT1) from Arabidopsis has been shown to be required for normal TAG accumulation in oil storing tissues, both by overexpression and mutation studies (Katavic et al., 1995; Routaboul et al., 1999; Jako et al., 2001; Zhang et al., 2009; Chapman and Ohlrogge, 2012). However, the function of DGAT2 in Arabidopsis remains unclear since the knockout mutant did not show a significant reduction of in seed oil content, even when crossed with a *dgat1* mutant (Liu et al., 2012). By contrast, DGAT2 from castor bean (*Ricinus communis*) endosperm, which accumulates a large amount of TAG containing more than 90% ricinoleic acid (18:1 FA hydroxylated at the Δ^12^-position), exhibits the highest expression levels in seeds and prefers acyl-CoA and DAG substrates containing hydroxylated FA (Burgal et al., 2008). Additionally, DGAT2 from tung tree (*Vernicia fordii*) producing TAGs enriched in α-eleostearic (18:3 *cis*Δ^9^, *trans*Δ^11^, *trans*Δ^13^) preferentially incorporates α-eleostearic acid into TAGs in seed oil bodies (Shockey et al., 2006). Moreover, in castor bean and tung tree, the expression levels of DGAT2 are higher than those of DGAT1 in developing seeds (Kroon et al., 2006; Shockey et al., 2006). Thus, DGAT2 appears to play a more prominent role than DGAT1 in the selective accumulation of unusual FAs in the seed oil of certain plants (Liu et al., 2012).

Oil palm (*Elaeis guineensis* Jacq.), which is a perennial monocotyledonous plant belonging to the Arecaceae palm family, is the most productive oil-bearing crop in the world (Bourgis et al., 2011). It accumulates nearly 90 and 60% oil contents in its mesocarp and kernel, respectively, with totally distinct FA profiles. The mesocarp contains only long-chain FAs, especially 16:0 and 18:1 (each about 40%), while the kernel possesses abundant medium-chain FAs, especially 12:0 (about 50%) (Siew et al., 1995; Corley and Tinker, 2003). Therefore, the study of oil accumulation in oil pericarp tissue cannot only explore the special molecular mechanism of high oil accumulation in oil palm mesocarp, but also enrich our molecular biological understanding of lipid metabolism regulation in different plant tissues. EgDGAT1-1 from oil palm has been demonstrated to prefer medium-chain FAs as substrates for TAG synthesis (Aymé et al., 2015). Given the role of DGAT2 can vary depending on the species, it is of great interest to elucidate the function of DGAT2 from oil palm mesocarp, which does not accumulate unusual oil. Here, we provide a detailed functional analysis of a DGAT2 from oil palm. First, the expression pattern of *EgDGAT2* was positively correlated with oil accumulation in the mesocarp during fruit development. Then, heterologous expression of *EgDGAT2* in mutant yeast H1246 restored TAG biosynthesis, and it showed a substrate preference for unsaturated FAs (16:1 and 18:1). Furthermore, seed-specific overexpression of *EgDGAT2* in Arabidopsis increased the content of polyunsaturated FAs (18:2 and 18:3) in seed TAGs. These results not only revealed the high oil accumulation in oil palm mesocarp, but also expanded potential candidates for oil metabolic engineering to modify the FA composition.

## Materials and Methods

### Plant Materials and Strains

Oil palm (*E. guineensis* Jacq.) fruits at five comparable developing stages [30–60 days after pollination (DAP) (phase 1), 60–100 DAP (phase 2), 100–120 DAP (phase 3), 120–140 DAP (phase 4), and 140–160 DAP (phase 5)] were harvested from Coconut Research Institute, Chinese Agricultural Academy of Tropical Crops, Hainan, China. The fruits were flash-frozen in liquid nitrogen a few minutes after harvest and stored at -80°C until use. After removal of the pericarp, mesocarp tissue samples were dissected and powdered in liquid nitrogen immediately for RNA extraction. Wild-type *Arabidopsis thaliana* ecotype Columbia was used in this study. Arabidopsis plants were grown in a growth chamber at 23°C with a 16-h photoperiod (16 h of 150 μE m^-2^ s^-1^ light and 8 h of darkness). The quadruple mutant yeast *Saccharomyces cerevisiae* strain H1246 that cannot synthesize TAGs (Sandager et al., 2002) was generously donated by Dr. Vincent Arondel from Laboratoire de Biogenèse Membranaire, Villenave-d’Ornon, France.

### RNA Extraction and cDNA Synthesis

Total RNA from oil palm mesocarp and Arabidopsis seeds was extracted using the cetyltrimethylammonium bromide (CTAB) method as described previously (Li and Fan, 2007). The quantity and quality of isolated total RNA were determined by spectrophotometry and gel electrophoresis, respectively. The total RNAs were used for cDNA synthesis, gene amplification, and expression analysis. First-strand cDNA was synthesized from 2 μg of total RNA from oil palm mesocarp and Arabidopsis seeds using TIANScript One Step RT-PCR Kits (Tiangen, Beijing, China), according to the manufacturer’s instructions. Reverse transcription was performed at 42°C for 1 h, with a final denaturation at 70°C for 15 min.

### Real-Time Quantitative PCR (RT-qPCR) Analysis

The cDNAs from Arabidopsis seeds or oil palm mesocarp at five different developmental stages were subjected to fluorescent quantitative RT-PCR to detect gene expression levels in different materials. Reactions were carried out in triplicate using the SYBR^®^ Premix Ex Taq^TM^ II (TaKaRa, Tokyo, Japan) and monitored with a CFX Connect^TM^ Real-Time (RT)-PCR Detection System (Bio-Rad, United States) according to the manufacturer’s instructions. The housekeeper genes Atβ-actin and Egβ-actin from Arabidopsis and oil palm were used as internal control genes. Primers used for RT-PCR were designed using the Primer Premier 5 program, and are listed in **Table 1** (RT-Atβ-actin F/R, RT-Egβ-actin F/R, and RT-EgDGAT2 F/R). The expression level of *EgDGAT2* was calculated in terms of comparative threshold cycle (Ct) using the 2^-ΔΔCt^ method (Livak and Schmittgen, 2001).

**Table 1:** Primers used in this study.

Name	Sequence (5′→3′)
RT-Atβ-actin F	TGGAAGCTGCTGGAATCCAT
RT-Atβ-actin R	TCCTCCACTGAGCACAACGTT
RT-Egβ-actin F	TGGAAGCTGCTGGAATCCAT
RT-Egβ-actin R	TCCTCCACTGAGCACAACGTT
RT-EgDGAT2F	TCCTAAGCCGACCATTGAA
RT-EgDGAT2R	CGTGACTGCCACTGATTTGA
5′RACE-EgDGAT2 R1	GCAACAACGCCAATCGGTAA
5′RACE-EgDGAT2 R2	CCTCCACATAAAGGGTCACAGG
pYES2-EgDGAT2F	TAGAATTCATGGACCACGGTAACGGA (*Eco*RI)
pYES2-EgDGAT2 R	GAGAGCTCTTATAGAACTCTCAAACGAAGATCA (*Sac*I)
1300s-EgDGAT2F	TAGGTACCATGGACCACGGTAACGGA (*Kpn*I)
1300s-EgDGAT2R	GAGGATCCTTATAGAACTCTCAAACGAAGATCA (*Bam*HI)


### Gene Cloning and Bioinformatics Analysis

To generate the coding sequence of *EgDGAT2*, rapid amplification of the cDNA ends (RACE) was performed using a BD Smart^TM^ RACE cDNA Amplification Kit (Clontech, United States). The primers for 5′-RACE (**Table 1**) were designed based on the sequence of *EgDGAT2* (Accession number: XM_010933834) from EST using the Primer Premier 5 program. All RACE products were purified with AxyPrep^TM^ DNA Gel Extraction Kit (Axygen, China) and cloned into PMD19-T Simple Vector (Takara, Japan) and fully sequenced by BGI Company (China).

The RACE products and original EST fragments were aligned and assembled into a full-length cDNA of *EgDGAT2* by the DNAMAN program. The open-reading frames (ORF) finder from NCBI^1^ was used to identify the ORF of *EgDGAT2*. The ORF of *EgDGAT2* was then amplified from oil palm cDNA using Q5 High-Fidelity DNA Polymerase (New England Biolabs, United Kingdom) and gene-specific primers pYES2-EgDGAT2 F/R (**Table 1**). The PCR conditions were as follows: 95°C for 3 min, 30 cycles of 94°C for 30 s, 55°C for 30 s, 72°C for 1 min, and 72°C for 7 min. The PCR product was cloned into vector pEASY-Blunt (TaKaRa, Japan) and sequenced.

The BLAST program from the NCBI website was used for nucleotide and amino acid sequence analyses. Protein topology was analyzed by TMHMM^2^. Amino acid alignment of DGATs was performed by Clustal Omega^3^ and a phylogenetic tree was constructed using the neighbor-joining method from MEGA 5.0 software (Tamura et al., 2011).

### Heterologous Expression of *EgDGAT2* in the Mutant Yeast H1246

*EgDGAT2* ORF fragments were digested with *Eco*RI and *Sac*I from the pEASY vector, and inserted into the yeast expression vector pYES2 which is a high-copy, autonomously replicated vector controlled by the inducible promoter *GAL1*. The construct of EgDGAT2-pYES2 was transformed into mutant yeast strain H1246 cells using the polyethylene glycol/lithium acetate method (Gietz et al., 1995). The H1246 cells transformed with empty vector pYES2 were used as the negative control. Transformants were selected on synthetic complete medium lacking uracil (SC-ura), supplemented with 2% (w/v) glucose. Three independent positive clones of each transformation were used for further expression studies.

Precultures were made in 2 mL SC-ura medium with 2% (w/v) raffinose, and were grown overnight at 30°C. The cells were washed with distilled water first and were diluted to a final optical density (OD_600_) of 0.4 in 20 mL induction medium SC-ura supplemented with 2% galactose. Then, the yeast cultures were incubated at 30°C for 24 h. Lastly, OD_600_ of the culture was recorded at least three times to precisely determine the quantity of yeast cells as OD units; 1 OD unit is the quantity of yeast cells that gives an OD_600_ of 1 in 1 mL. The cells were harvested by centrifugation and washed three times with distilled water for lipid analysis.

### Plant Transformation and Selection

To generate a binary plant overexpression construct, the ORF of EgDGAT2 was re-amplified from positive construct EgDGAT2-pYES2 using primers 1300s-EgDGAT2 F/R (**Table 1**). Then, the PCR product was purified and digested with *Kpn*I and *Bam*HI, and ligated into binary expression vector pCAMBIA1300s under the control of a seed-specific napin promoter to generate the plant expression construct EgDGAT2-pCAMBIA1300s. Subsequently, it was introduced into *Agrobacterium tumefaciens* strain GV3101 by electroporation and used to transform wild-type *A. thaliana* (ecotype Columbia) by the floral dip method (Clough and Bent, 1998). Transformants were selected on Murashige and Skoog (MS) medium containing 30 mg/L hygromycin B (Sigma), and then confirmed by PCR of genomic DNA from leaves using primers 1300s-EgDGAT2 F/R (**Table 1**). Homozygous lines were identified in T_3_ generation by segregation analyses.

### Lipid Extraction and Separation by Thin Layer Chromatography (TLC)

Lipid extraction from yeast cells was performed using method described before (Dittrich-Domergue et al., 2014). Then, the total lipids were dissolved in chloroform and used for thin layer chromatography (TLC) to separate the neutral lipids on TLC silica Gel 60 plates. The solvent system used for development was hexane/diethyl ether/acetic acid (80:20:2, v/v/v). Lipid spots were revealed by spraying the plates with 0.01% primuline in acetone/H_2_O (60:40; v/v) and visualized under ultraviolet light at 366 nm. TAG spots were scratched from the plate for making fatty acid methyl esters (FAMEs) and gas chromatography (GC) analysis. An appropriate amount of internal standard TAG 17:0 was mixed with samples loaded on the plate for TAG quantification.

Fatty acid methyl esters were derived using 2 mL of methanol that contained 2.5% sulfuric acid (v/v). The mixture was incubated at 80°C for 2 h, after which 2 mL of 0.9% NaCl (w/v) and 3 mL hexane were added. The mixture was homogenized and centrifuged to separate the layers. The upper organic phase was transferred into a new glass tube, and used for GC analysis as described in Yuan et al. (2015).

Total lipids were extracted from Arabidopsis seeds using chloroform/methanol (2:1, v/v), and resuspended in chloroform for subsequent methyl esterification, GC and TLC analyses as described previously. An appropriate amount of FA 17:0 was added as internal standard for quantification of total lipids.

### Statistical Analysis

Three biological replicates were used for every sample to ensure reproducibility, and the data provided as mean ± standard deviation. Statistical analyses were performed using Statview 6.0 (SAS Institute, Cary, NC, United States) and Microsoft Office Excel 2007 (Microsoft Corporation, Redmond, WA, United States) to obtain intergroup comparisons between two groups used a paired *t*-test. *P*-value < 0.05 was considered to indicate a statistically significant difference. *P* ≤ 0.01 was regarded as highly significant, and a difference with *P* ≤ 0.001 was extremely significant.

## Results

### Isolation and Identification of a Putative *DGAT2* from Oil Palm

The full-length cDNA (999 bp) of a putative *DGAT2* was isolated from oil palm mesocarp, thereafter designated as *EgDGAT2*, based on the partial sequence of DAG *O*-acyltransferase 2 from *E. guineensis* (accession number: XM_010933834) and the fragments from 5′-RACE results. It encoded a predicted polypeptide of 332 amino acids with 37.28 kD mwt and a PI of 9.53, which contains two putative transmembrane domains close to the N-terminus, at amino acid residues 32–54 and 56–78. Bioinformatics analysis of EgDGAT2 indicated that it possesses the conserved domain of DAG *O*-acyltransferase (PLN02783; amino acids 24–332), which is involved in the terminal step of TAG synthesis.

**FIGURE 1:**
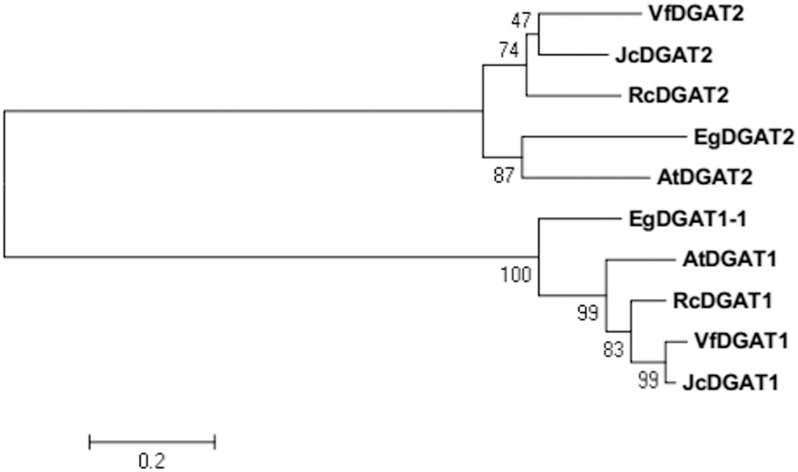
Phylogenetic analysis of EgDGAT2 and Type 2 and Type 1 DGATs from other species. Phylogenetic tree was generated by the neighbor-joining method in MEGA5. The GenBank accession numbers of the sequences showed are as follows: EgDGAT1-1 (XP_010924968.1) from *Elaeis guineensis*, AtDGAT1 (NP_179535.1) and AtDGAT2 (NP_566952.1) from *Arabidopsis thaliana*, RcDGAT1 (NP_001310663.1) and RcDGAT2 (NP_001310616.1) from *Ricinus communis*, JcDGAT1 (ABB84383.1) and JcDGAT2 (AFV61670.1) from *Jatropha curcas*, and VfDGAT1 (ABC94471.1) and VfDGAT2 (ABC94473.1) from *Vernicia fordii*.

**FIGURE 2:**
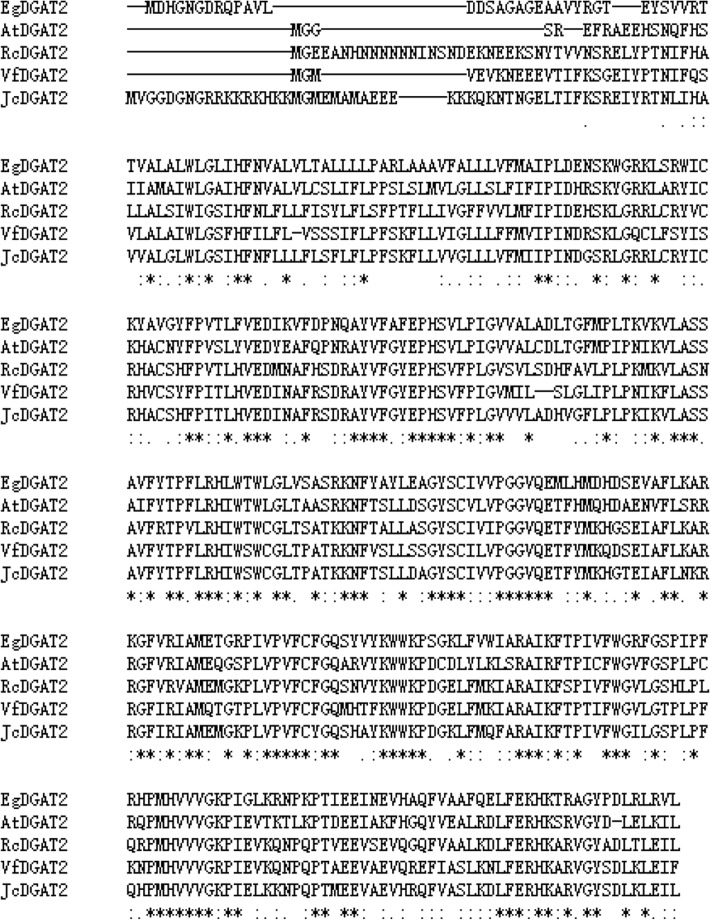
Alignment of deduced amino acid sequence of EgDGAT2 with selected plant homologs by Clustal Omega. The GenBank accession numbers of the sequences showed are as follows: AtDGAT2 (NP_566952.1) from *Arabidopsis thaliana*, RcDGAT2 (NP_001310616.1) from *R. communis*, JcDGAT2 (AFV61670.1) from *J. curcas*, and VfDGAT2 (ABC94473.1) from *V. fordii*.

BLAST analysis showed that EgDGAT2 shared high homology with DGAT2 from other plants, such as 62% identity/94% similarity with AtDGAT2 (NP_566952.1). To elucidate phylogenetic relationships of *EgDGAT2*, the deduced amino acid sequence was aligned with Type 2 and Type 1 DGATs from other higher plants and a neighbor-joining tree was constructed (**Figure 1**). There were two distinct branches representing the two types of DGAT families in the tree, and EgDGAT2 clustered in the DGAT2 branch (**Figure 1**). Moreover, EgDGAT2 was grouped with AtDGAT2 (**Figure 1**). Then, multiple sequence alignment of EgDGAT2 and other DGAT2s from several different plants was carried out. As shown in **Figure 2**, EgDGAT2 shared high identities with DGAT2 proteins from other plant species, including AtDGAT2 (NP_566952.1) from *A. thaliana*, RcDGAT2 (NP_001310616.1) from *R. communis*, JcDGAT2 (AFV61670.1) from *Jatropha curcas*, and VfDGAT2 (ABC94473.1) from *V. fordii*.

### Analysis of the Spatial Expression of *EgDGAT2* in Mesocarp during Fruit Development

**FIGURE 3:**
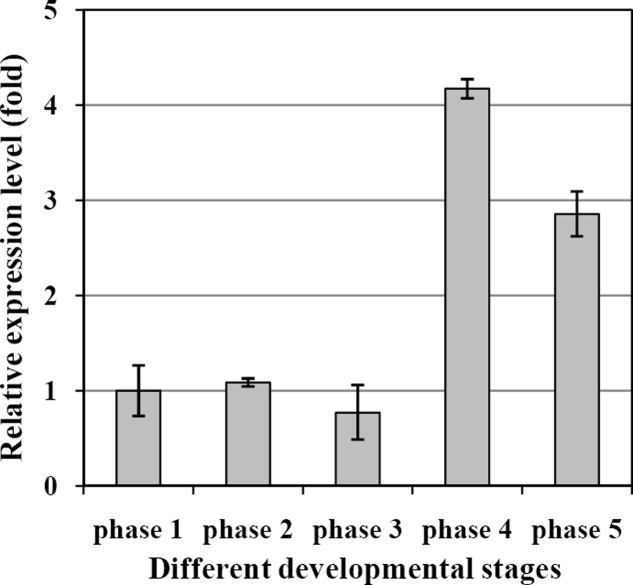
Temporal expression patterns of *EgDGAT2* in oil palm mesocarp at five different developmental stages: phase 1: 30–60 DAP; phase 2: 60–100 DAP; phase 3: 100–120 DAP; phase 4: 120–140 DAP; and phase 5: 140–160 DAP.

The transcript levels of *EgDGAT2* in mesocarp at five different developmental stages were determined by RT-qPCR using β-actin as an internal control. The results showed that the expression level of *EgDGAT2* was rather flat during the first three developmental stages (phases 1–3), then significantly increased up to fourfold in phase 4, and slightly decreased by 25% during the fruit ripening phase (phase 5) (**Figure 3**).

### Heterologous Expression of *EgDGAT2* Restores TAG Biosynthesis in the Yeast TAG-Deficient Mutant H1246

To investigate the *in vivo* function of EgDGAT2, we first performed the heterologous expression of *EgDGAT2* in the quadruple mutant yeast H1246, which is devoid of neutral lipids (Sandager et al., 2002). An H1246 strain transformed with an empty pYES2 vector was used as a negative control. After 48 h of induction, the yeast cells were collected for lipid extraction. Then, neutral lipids were separated by TLC using hexane/diethyl ether/acetic acid (80:20:2; v/v/v) as the mobile phase. The result revealed that the transformants expressing *EgDGAT2* had significant spots corresponding to TAGs, while TAGs were not detected in the negative control cells carrying the empty expression vector (data not shown), as expected. Thus, EgDGAT2 restored TAG synthesis in yeast, which suggested that *EgDGAT2* encoded a protein with DGAT activity.

**FIGURE 4:**
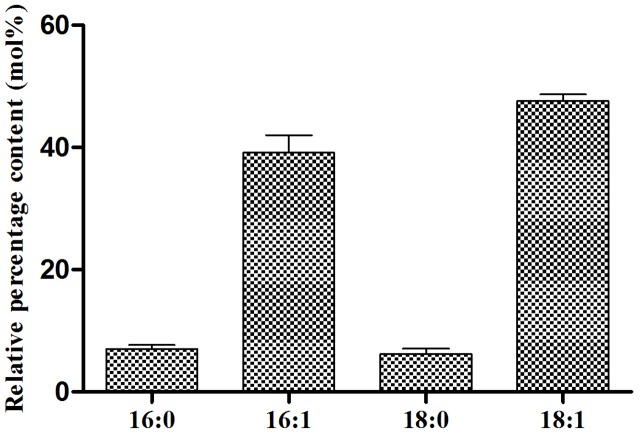
Fatty acid composition of triacylglycerol from *S. cerevisiae* H1246 strain expressing *EgDGAT2*. Neutral lipids from yeast cells were separated by TLC and the TAG spot was scraped from the plate, and used for transmethylation and GC analysis. Fatty acid abbreviations are as follows: 16:0, palmitic acid; 16:1, palmitoleic acid; 18:0, stearic acid; 18:1, oleic acid.

In order to elucidate the FA profile of TAGs EgDGAT2 produced in yeast, the transmethylation of TAGs was performed, and GC analysis was carried out. The negative control we mentioned before could not synthesize TAGs. As shown in **Figure 4**, 16:1 and 18:1 were two major FAs, accounting for 36.21 and 48.65%, respectively. Meanwhile, there were two minor FAs: 16:0 and 18:0 (each about 6%) (**Figure 4**). Thus, EgDGAT2 produces TAGs with rich unsaturated FAs (up to 85%) in yeast.

### Heterologous Expression of *EgDGAT2* in *Arabidopsis thaliana*

In order to further verify the *in vivo* activity of EgDGAT2 *in planta*, *EgDGAT2* was cloned into pCAMBIA1300s-napin vector driven by a seed-specific promoter napin. The recombinant plasmid was introduced into *A. tumefaciens* and used to transform wild-type Arabidopsis. The transgenic lines were first selected by hygromycin in 1/2 MS medium, then transplanted to soil until maturity. Five positive transgenic plants were selected and confirmed by PCR using genomic DNA from T_1_/T_2_ transgenic Arabidopsis seedlings as template and primers 1300s-EgDGAT2 F/R (**Table 1**).

**FIGURE 5:**
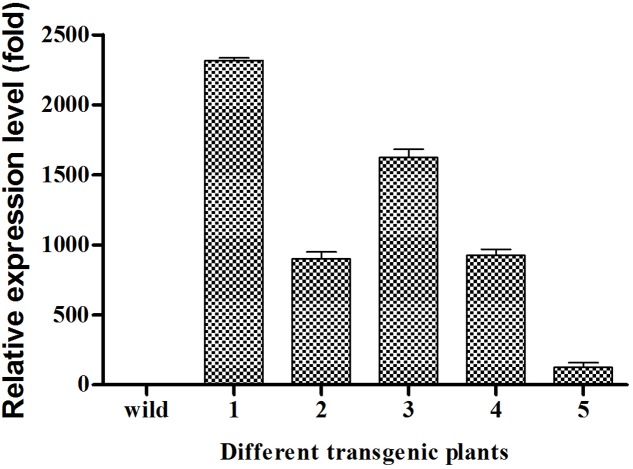
Expression levels of *EgDGAT2* in transgenic *Arabidopsis thaliana* seeds.

The transcript levels of *EgDGAT2* in several T_3_ homozygous transgenic Arabidopsis seeds were quantified by RT-qPCR. The results showed that *EgDGAT2* transcript expression in seeds varied up to 2300-fold between different transgenic lines, with two of them (1 and 3) exhibiting over 1500-fold *EgDGAT2* expression compared with the wild-type, and two of them (2 and 4) around 1000-fold *EgDGAT2* expression compared with the wild-type (**Figure 5**). Three transformant lines (2, 3, and 5) with different levels of *EgDGAT2* transcript were selected for further analysis.

### Seed-Specific *EgDGAT2* Overexpression Enhances Polyunsaturated Fatty Acid Content in Arabidopsis Seeds

**FIGURE 6:**
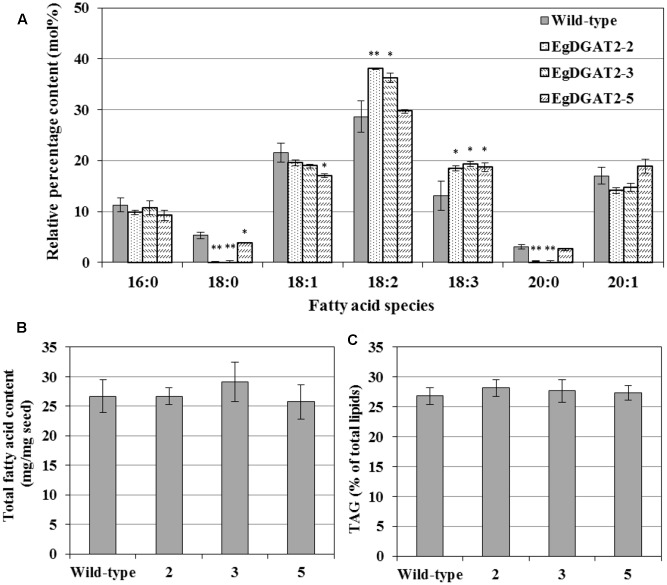
Fatty acid composition of TAG and lipid content from wild-type and *EgDGAT2* transgenic Arabidopsis seeds: **(A)** fatty acid composition of TAG; **(B)** Total fatty acid content of Arabidopsis seeds (dry weight); **(C)** TAG proportion of total lipids in Arabidopsis seeds. EgDGAT2-2, 3, and 5 were three individual transgenic Arabidopsis plants. Fatty acid abbreviations are as follows: 16:0, palmitic acid; 18:0, stearic acid; 18:1, oleic acid; 18:2, linoleic acid; 18:3, linolenic acid; 20:0, eicosanoic acid; 20:1, eicosenoic acid.

To investigate the effects of overexpression of *EgDGAT2* in Arabidopsis seeds, the relevant products of EgDGAT2 were analyzed, including FA composition of TAGs, total FA content, and TAG content. Total lipids of mature seeds from homozygous T_3_ transgenic lines and wild-type Arabidopsis were extracted, and neutral lipids were separated by TLC, transmethylated, and analyzed by GC as described previously. As shown in **Figure 6A**, the EgDGAT2 transgenic seeds contained 34.7 mol% 18:2 FA on average, versus 28.66 mol% in wild-type seeds. In addition, the content of 18:3 FA in seed TAGs from EgDGAT2 over-expression lines was increased from 13.1 up to 18.8 mol% when compared with wild-type seeds (**Figure 6A**). By contrast, the levels of 18:0 and 20:0 FA in *EgDGAT2* transgenic lines were significantly decreased to 1.4 and 1.0 mol%, compared with 5.3 and 3.1 mol% in seed TAGs from wild-type lines, respectively (**Figure 6A**). There was no significant difference in the levels of other FAs when TAG seeds from transgenic and wild-type Arabidopsis plants were compared (**Figure 6A**). Moreover, no obvious difference was observed in the total FA content and TAG content when seeds from transgenic and untransformed Arabidopsis plants were compared (**Figures 6B,C**).

## Discussion

In the present study, we identified and characterized one *EgDGAT2* from oil palm mesocarp. When heterologously expressed in mutant yeast H1246, it restored TAG biosynthesis and exhibited a preference for unsaturated FA 16:1 and 18:1. Moreover, the specific overexpression of *EgDGAT2* in Arabidopsis seeds led to an increase in polyunsaturated FAs (18:2 and 18:3) in seed TAGs. These results increase the understanding of the *in vivo* activities of EgDGAT2 from oil palm in yeast and plants.

While at least four classes of DGATs have been identified based on structure and activity, only the two most common types, DGAT1 and DGAT2, are well-understood. EgDGAT2 contains two predicted transmembrane domains, which were adjacent and located near the N-terminus. Moreover, it also possesses conserved and functional motifs of DGAT2 (**Figure 2**). All these characteristics of the protein sequence strongly indicated this protein EgDGAT2 belongs to DGAT2 family, rather than DGAT1 (Liu et al., 2012). Furthermore, the evolutionary analysis supported this hypothesis as EgDGAT2 clustered with DGAT2 proteins from other plant species, especially AtDGAT2 from Arabidopsis (**Figure 1**).

From the expression pattern during fruit development, we can see *EgDGAT2* showed higher expression level at the fourth and fifth stages (**Figure 3**), while large amounts of oil were deposited in the mesocarp (Bourgis et al., 2011; Tranbarger et al., 2011). Thus, expression of *EgDGAT2* is closely correlated with oil deposition in oil palm mesocarp. Similar patterns are seen in the expression of *DGAT2* and oil accumulation in tung tree and castor bean (Shockey et al., 2006; Cagliari et al., 2010), whereas *DGAT2* in soybean and Arabidopsis shows very limited expression in seeds (Liu et al., 2012). Moreover, seeds from tung tree and castor bean both accumulate the unusual FAs, α-eleostearic acid and ricinoleic acid, respectively (Shockey et al., 2006; Burgal et al., 2008). Since the two major FAs in the oil palm mesocarp are 16:0 and 18:1, approximately 40% in each case, we hypothesized that EgDGAT2 might be responsible for TAGs rich in these unusual FAs: 16:0 and 18:1. Subsequently, *in vivo* experiments of EgDGAT2 in mutant yeast and plants were conducted to analyze their function and substrate preference.

As predicted, EgDGAT2 restored TAG biosynthesis in mutant yeast H1246. The resulting TAGs were enriched in saturated FAs (85%), indicating that EgDGAT2 had a marked substrate preference for unsaturated FAs, when compared with the wild-type SCY62 transformed with empty vector pYES, which had about 68% saturated FA (unpublished data). Similarly, AtDGAT2 from Arabidopsis and GmDGAT2D from soybean (*Glycine max*) both restored neutral lipid synthesis and exhibited substrate preference for unsaturated FA when expressed in mutant yeast H1246 (Aymé et al., 2014; Chen et al., 2016). However, unlike AtDGAT2 which has a very limited expression level during seed development (Liu et al., 2012), *EgDGAT2* transcripts were positively correlated with oil accumulation in oil mesocarp during fruit development (**Figure 3**). Taken together, it suggested EgDGAT2 might play an important role in oil deposition occurring in oil palm mesocarp.

Moreover, the heterologous seed-specific expression of *EgDGAT2* in Arabidopsis led to the increase of polyunsaturated FAs (18:2 and 18:3) esterified to TAGs in seeds (**Figure 6A**). Likewise, overexpression of *GmDGAT2D* in Arabidopsis seeds also increased the amount of 18:2 in TAGs, although the content of 18:3 was reduced (Chen et al., 2016). However, other systems expressing DGAT2 have different FA composition, for example, TAGs in the oil palm mesocarp comprised only 10% 18:2 FA, and contained no 18:3 FA (Sambanthamurthi et al., 2000), and *S. cerevisiae* contains no 18:2 or 18:3 FAs. Clearly, EgDGAT2 prefers to use unsaturated FAs as substrates in both yeast and plant.

## Conclusion

These results increase our understanding of oil synthesis in the oil palm mesocarp, which might be of importance for modifying FA composition as desired in oil metabolic engineering. For instance, coexpression *EgDGAT2* with specific FA desaturase (*FAD2* or *FAD3*) or other genes encoding enzymes involved in complex acyl-editing pathways might further increase the proportion of polyunsaturated FAs in seed TAGs. However, no *in vivo* system including a medium FA profile was tested in this paper. Given that abundant medium-chain FAs are accumulated in oil palm kernel oil (Siew et al., 1995), whether EgDGAT2 possesses the capability to use medium-chain acyl-CoAs as substrates still remains unknown. In addition to EgDGAT1-1 and EgDGAT2, there might be other DGAT homologs which also make a contribution to TAG accumulation in oil palm fruits (Dussert et al., 2013). Overall, deciphering the *in vivo* activity of EgDGAT2 enzyme from oil palm will contribute to oil metabolic engineering in generating preferential oil composition in common crops.

## Author Contributions

DL and YZ designed the research. YJ, YY, LG, RS, and LC performed the research. YJ and YY wrote the paper. All authors read and approved the final manuscript.

## Conflict of Interest Statement

The authors declare that the research was conducted in the absence of any commercial or financial relationships that could be construed as a potential conflict of interest.

## Footnotes

1 http://www.ncbi.nlm.nih.gov/gorf/gorf.html

2 www.cbs.dtu.dk/services/TMHMM/

3 http://www.ebi.ac.uk/Tools/msa/clustalo/
